# Inflamed Appendix Protruding Through a Right Broad Ligament Defect: A Case Report

**DOI:** 10.7759/cureus.65252

**Published:** 2024-07-24

**Authors:** Elissavet Symeonidou, Ioannis Gkoutziotis, Maria S SidiropouIou, Chrysoula Gouta, Kalliopi Gianna, Konstantinos Mpallas

**Affiliations:** 1 5th Department of Surgery, Ippokrateio General Hospital of Thessaloniki, Thessaloniki, GRC; 2 School of Medicine, Aristotle University of Thessaloniki, Thessaloniki, GRC; 3 Department of Radiology, Ippokrateio General Hospital of Thessaloniki, Thessaloniki, GRC; 4 Department of Pathology, Ippokrateio General Hospital of Thessaloniki, Thessaloniki, GRC

**Keywords:** laparoscopy, acute abdomen, neuroendocrine tumor, broad ligament defect, appendicitis

## Abstract

The protrusion of the appendix through a broad ligament defect suggests an extremely rare clinical entity, especially when the clinical manifestation of this condition is acute appendicitis. The current article presents a case of a 23-year-old female who presented to the emergency department with right lower abdominal pain and nausea, accompanied by elevated white blood cell count. Imaging performed, both ultrasound and computed tomography, reported acute appendicitis, without evidence of a broad ligament defect. During exploratory laparoscopy, the defect was identified, the appendix was retrieved and ligated and sutures were applied to the defect. The postoperative course of treatment was uneventful, and pathology confirmed inflammation of the appendix and also revealed a small neuroendocrine tumor. The value of emergency laparoscopy for the diagnosis of this rare condition and the therapeutic management which involves the closure of the defect to prevent future internal hernia are issues highlighted in this article.

## Introduction

Broad ligament defects are sporadic as they account for 4-7% of internal hernias, which suggests less than 1% of all hernias [1,2]. They can be congenital or acquired [3], unilateral or bilateral [1], and sometimes accompanied by other abnormalities [1]. Usually, they cause small bowel obstruction, which might lead to strangulation of the bowel [3], although sigmoid herniation has also been reported [4]. A simple classification of broad ligament defects has been proposed by Cilley et al. [5], based on anatomic landmarks. Their association with acute appendicitis has not been described in the literature and only a couple of articles report the protrusion of the appendix through a broad ligament defect [1]. This atypical location of the appendix cannot be obvious with certainty in preoperative imaging because of the rarity of this condition [3], although imaging can indicate close proximity with the ovary or the uterus [6]. In addition, conventional open appendicectomy would not reveal this anatomic deformity. However, the increasing application of laparoscopic appendicectomy, which has become the gold standard of acute appendicitis treatment, might lead the surgeon to new challenges. Laparoscopy enables not only the diagnosis but also the treatment even for broad ligament hernias requiring small bowel resection, thus avoiding the disadvantages of a laparotomy [7].

In this article, we present the case of a 23-year-old woman who presented to the Emergency Department complaining of right lower abdominal pain of acute onset. Her white blood cells were elevated and imaging, both ultrasound and computed tomography, suggested signs of acute appendicitis. Laparoscopy revealed the protrusion of the appendix through a broad ligament defect. Laparoscopic appendicectomy followed by closure of the defect was conducted. Her postoperative course of treatment was uneventful. The pathology report confirmed acute appendicitis and revealed a small neuroendocrine tumor (NET). This article describes a unique case with acute appendicitis which protrudes through a broad ligament defect.

## Case presentation

A 23-year-old Caucasian female presented to the Emergency Department complaining of abdominal pain of acute onset, located in the right iliac fossa toward the hypogastrium. The pain lasted a few hours and was accompanied by nausea. No history of previous abdominal surgery or any health problems was reported. She mentioned no allergies and she was a nonsmoker. There was nothing remarkable concerning her family medical history.

**Figure 1:**
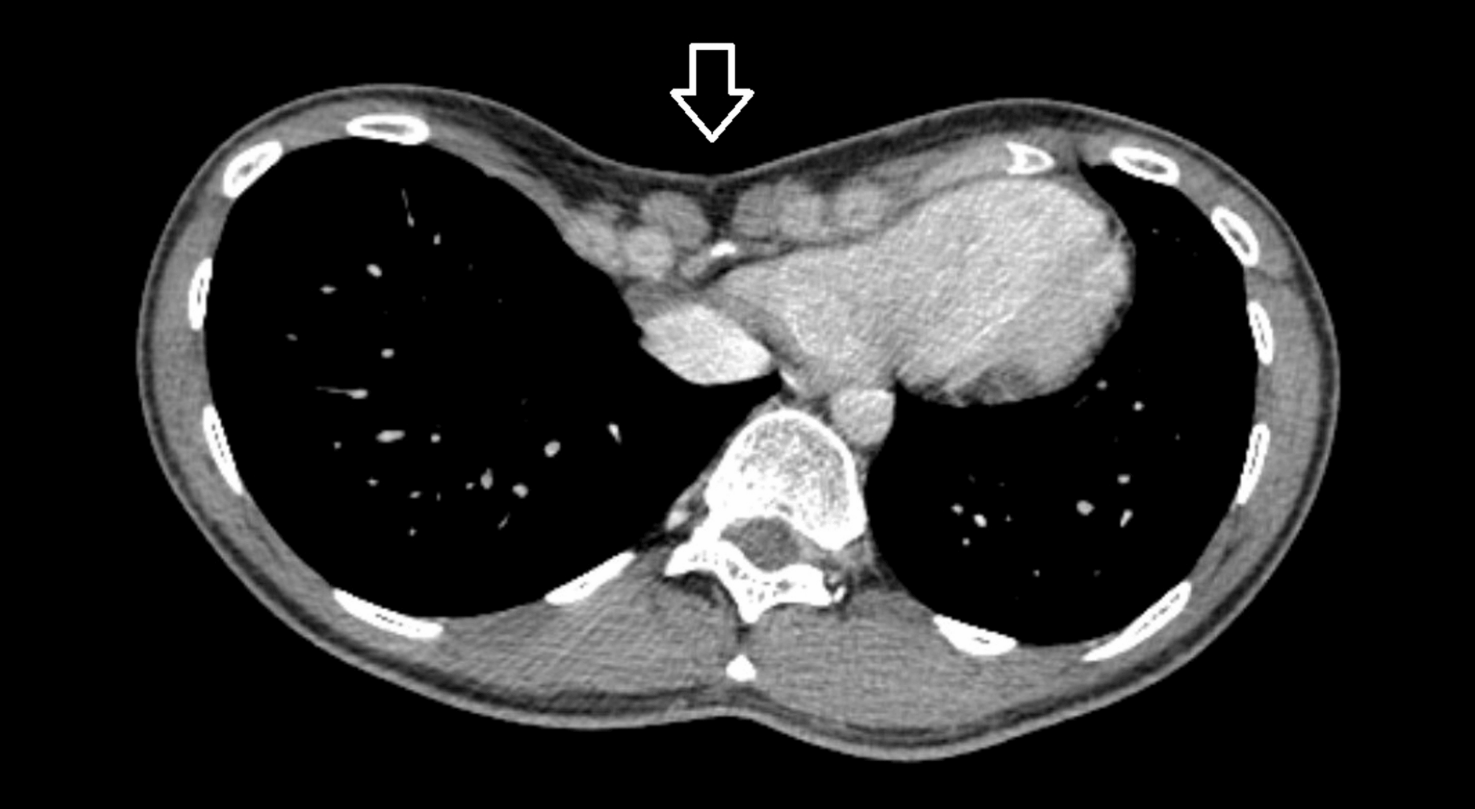
Computed tomography imaging with intravenous contrast Pectus excavatum (white arrow)

Upon admission, the patient was afebrile and her vital signs were stable; the blood pressure was 112/70 mm Hg with 72 heartbeats per minute, 100% oxygen saturation, and temperature 36.5 degrees Celsius. The clinical examination revealed tenderness at the right lower quadrant toward the hypogastrium and positive McBurney and Blumberg signs. In addition, pectus excavatum, a congenital chest wall deformity, was noticed (Figure 1). However, no further investigation was done and no specific anesthetic considerations were taken regarding this deformity.

Regarding laboratory investigation, white blood cells were elevated, 13500 x 10^3 /μL (3800-10500x10^3), with 83% neutrophils, but C-reactive protein was within the normal range; 3.6 mg/L (<6 mg/L). The rest of the blood and urine exams were normal, and the pregnancy test was negative.

**Figure 2:**
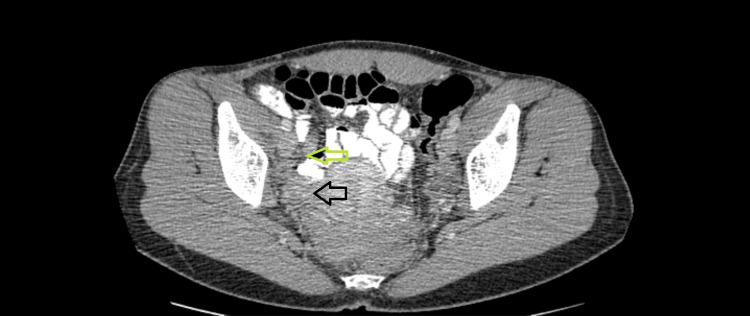
Computed tomography imaging of the abdomen, with intravenous and oral contrast The appendix (green arrow) with signs of inflammation, located next to the right ovary (black arrow)

**Figure 3:**
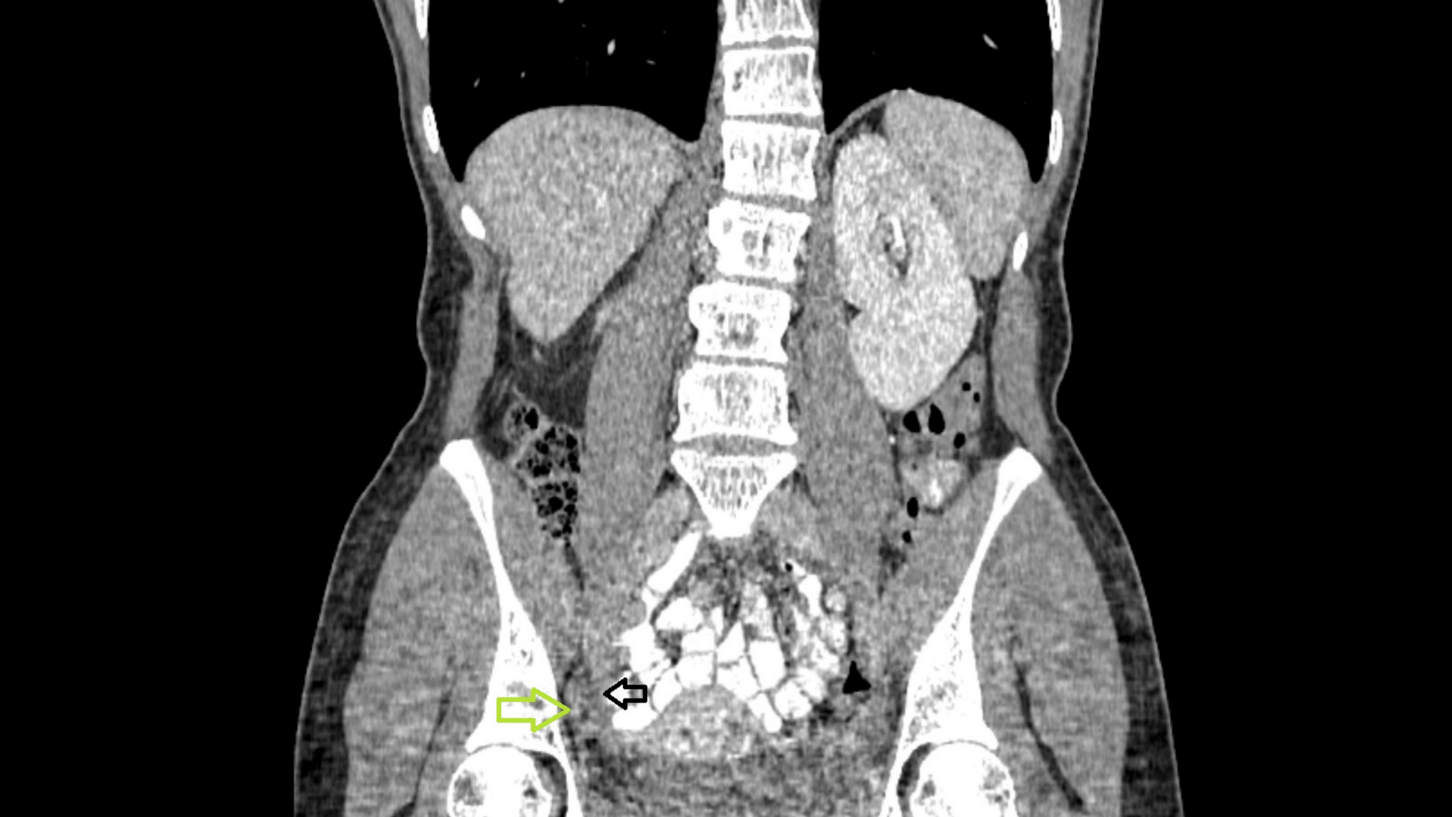
Computed tomography imaging with intravenous and oral contrast in coronal view The close association between the appendix (green arrow) and the broad ligament (black arrow)

Ultrasound of the right lower abdomen suggested acute appendicitis, with 11mm dilation of the appendix, without intraabdominal fluid. However, because of the acute onset of the pain and the normal CRP, it was decided to proceed with a computed tomography (CT) scan. A dilated appendix, 12 mm in width and 7.2 cm in length, accompanied by edema and stranding of the periappendiceal fat was noticed, located next to the right adnexa (Figure 2) and the broad ligament (Figure 3).

**Figure 4:**
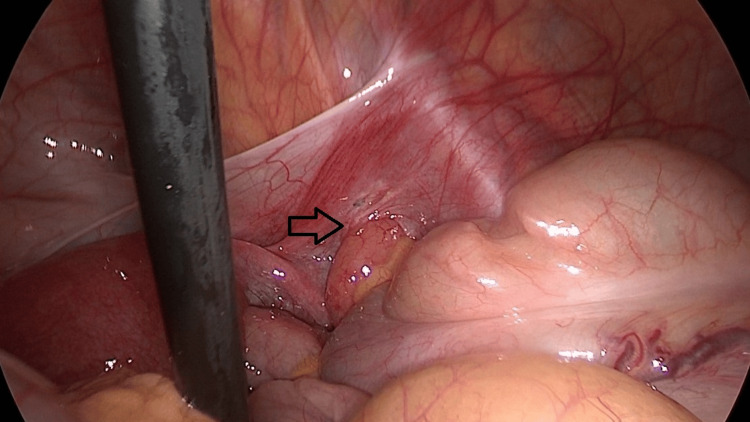
Intraoperative image during laparoscopy The appendix is protruding through a right broad ligament defect (black arrow)

**Figure 5:**
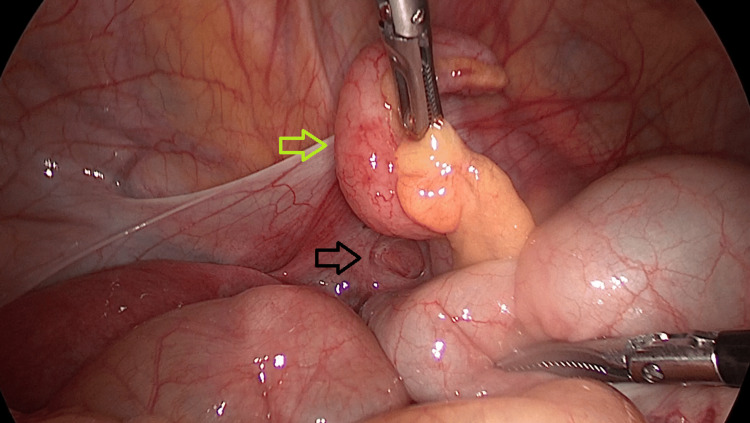
Intraoperative image during laparoscopy The right broad ligament defect (black arrow) and the appendix with macroscopic signs of acute appendicitis (green arrow)

The patient underwent an exploratory laparoscopy, which revealed the inflamed appendix herniated inside a defect in the right broad ligament, as shown in Figures 4, 5. A laparoscopic appendicectomy was performed, and interrupted nonabsorbable braided 2-0 sutures were placed to close the defect, which was approximately 2.5 cm in size. The left side was explored and the presence of a contralateral broad ligament defect was ruled out. Her postoperative stay was uneventful and she was discharged on postoperative day 1.

**Figure 6:**
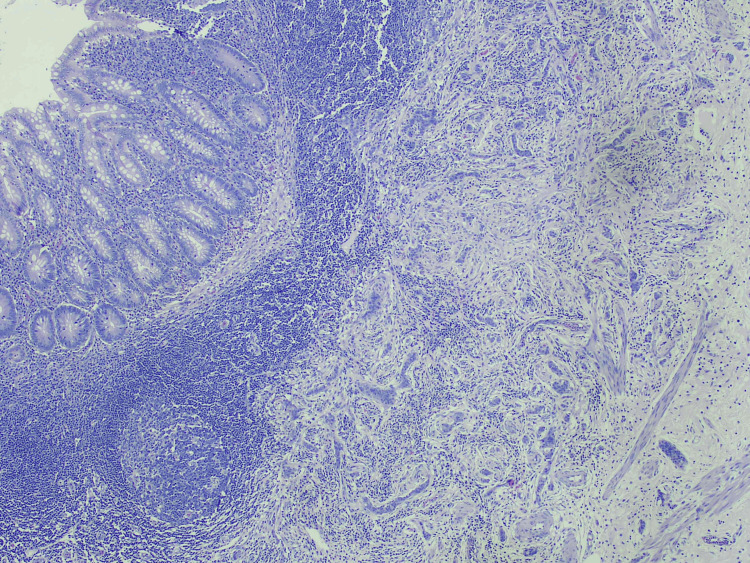
Pathology report Hematoxylin eosin staining, 4x, with apparent signs of inflammation

Histopathological examination reported signs of acute appendicitis and an incidental NET, 8 mm in size, well-differentiated, grade 1 (ki-67<3%) located in the apex of the appendix, T1 (Figure 6).

**Figure 7:**
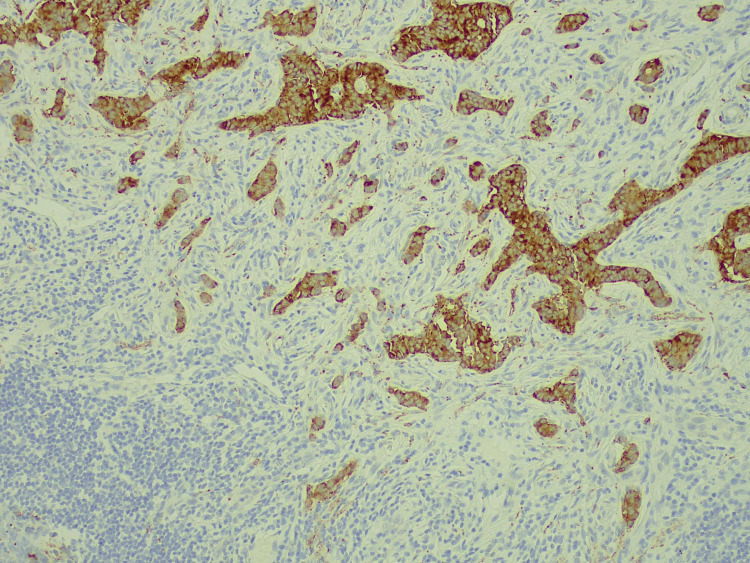
Immunohistochemistry Synaptophysin staining positive

Immunohistochemistry was positive for chromogranin and synaptophysin (Figure 7).

Multidisciplinary team meeting decided on observation including clinical and laboratory examination every six months, and annual CT of the abdomen. Six months later, the patient is disease-free. 

## Discussion

Broad ligament defects are extremely rare, as they account for 4-7% of internal hernias, which suggests less than 1% of all hernias [1,2]. They may be congenital or acquired, following a surgical, obstetric, or gynecological procedure, endometriosis [3], inflammatory pelvic disease [2], or placental abruption [4]. Congenital broad ligament defects might be accompanied by other conditions, such as phocomelia [1]. Rupture of cystic structures inside the broad ligament, which are remnants of the mesonephric of the Mullerian ducts, has been proposed as a pathophysiological mechanism [2]. They are usually unilateral, although a few cases of bilateral presentation have been reported. They are also classified into two types: the fenestra type, which involves complete two-layer deficiency, and the pouch type, in which the herniation happens toward the broad ligament from the anterior opening [2]. Cilley’s classification is another one based on anatomic marks and classifies broad ligament hernias into three types; type 1 where the defect is located caudal to the round ligament, type 2 where the defect is found above the round ligament between the mesovarium and the mesosalpinx, and type 3 where the defect is located in the mesoligamentum teres of the uterus [5]. Our patient had a unilateral congenital broad ligament pouch-type defect on the right side, in the absence of any previous operations, without any other coexisting anomalies.

Neither abdominal ultrasound nor CT can reveal the defect. Diagnostic laparoscopy is the best investigation [1]. Plain radiographs and CT scans can show signs of bowel obstruction, while CT can suggest the presence of an internal hernia and point out the level of obstruction inside the pelvis and next to the uterus [6,7]. In our case, although both ultrasound and CT set the diagnosis of acute appendicitis, none of them identified the broad ligament defect, which was diagnosed during laparoscopy.

Overall ileum is the most common incancerated viscera [6], leading to internal hernia and mechanical bowel obstruction, which suggests the main clinical presentation of a broad ligament defect. The appendix, the cecum, and the small bowel [8] might be herniated through a right broad ligament defect, while the small bowel and the sigmoid colon are most likely herniated on the left side. The presence of a long sigmoid and its weak fixation to the retroperitoneum increase the possibility of sigmoid herniation [9]. Parts of the adnexa might also become herniated. The protrusion of two different structures such as the ileus and the fallopian tube has also been reported [10]. Pregnancy has been proposed as a risk factor [1], due to higher intraabdominal pressure. If the appendix is the herniated viscera, the arterial blood flow gets compromised and in combination with the venous obstruction, acute appendicitis is the direct consequence [1]. Increased length of the appendix, lower location of the cecum in the pelvis, and a large mobile cecum that pushes the appendix in the pelvis have been proposed as predisposing factors for the herniation of the appendix in cases of De Garengeot hernias [11] and might be similar to the herniation of the appendix in a broad ligament defect, although more data are needed. On the other hand, previous appendectomy has been reported in two cases prior to small bowel herniation caused by a broad ligament defect [6,8].

Only a few cases of acute appendicitis herniated in a broad ligament defect are reported in the literature, highlighting the clinical significance of this case presentation. Thorough research in PubMed, Scopus, and Google Scholar was conducted using the keywords <append*> AND <broad ligament>. The first case mentioned was in 1950 by Delannoy et al. [12], in a patient with gynecologic malignancy. Onida et al. [1] published a case report with the incarcerated cecum and appendix through the broad ligament defect. No other case with acute appendicitis and a broad ligament defect was found. Although small bowel herniation and ileus due to broad ligament defects are more frequently described, the protrusion of the appendix with concomitant acute appendicitis seems extremely rare. It is possible that with the wider application of exploratory laparoscopies, more such cases are discovered.

There is a possibility that salpingo-oophorectomy may be inevitable during surgery, although it is unnecessary in the majority of cases [1,13]. However, there is a risk of re-herniation when left behind, especially because of their small size. Open appendicectomy in these cases requires a wider incision for the complete visualization of the operation field and the defect repair. For this reason, the laparoscopic approach is the standard of care not only for the diagnosis but also for operative management. It offers a complete reduction of the hernia, closure of the defect, and careful inspection of the abdominal viscera, in combination with minimal surgical trauma and postoperative pain [2]. Even if conversion to open is necessary due to incarcerated viscera, previous laparoscopy allows a smaller incision [10]. Nasogastric tube placement is recommended prior to laparoscopy when ileus is present.

The closure of a uterine broad ligament defect is recommended since it is likely to cause herniation if left unsutured [1]. Simple 2-0 or 3-0 nonabsorbable braided sutures may be used for the defect closure [2,7]. The dissection of the broad ligament, both the anterior and posterior peritoneal layers, has also been proposed, as an easier solution, especially for postmenopausal patients [6]. However, simple closure is performed more frequently according to the literature [10]. The inspection of the contralateral broad ligament during laparoscopy is essential because of the possibility of bilateral presentation [10].

Incidental NETs are detected in 0.3-0.9% of the histopathology specimens following appendectomy, while the tip of the appendix is the most common location [14]. Acute appendicitis is the initial clinical manifestation of primary malignancies of the appendix in more than 50% of the cases [15]. In patients presenting with appendiceal inflammatory mass, the rate of tumor involvement reaches 10 to 29% [15]. For NETs with a diameter of less than 1 cm, located in the tip of the appendix, like in the above case, appendectomy is the standard surgical procedure [14]. 

Broad ligament defects should be added in the differential diagnosis of acute abdomen in females, not only for small intestine herniation but also for acute appendicitis, especially when it is combined with atypical clinical presentation. The association of this clinical condition with pectus excavatum and NETs, conditions that both co-existed in this case, is unknown.

## Conclusions

The possibility of a broad ligament defect should be considered in the differential diagnosis for female patients presenting with acute appendicitis, with or without prior operations. History of previous gynecologic procedures, obstetric surgery, inflammatory pelvic disease, or intestinal obstruction next to the uterus should raise further suspicions. Closure of the defect is important in order to prevent future internal hernias. Last but not least, the value of laparoscopy for the management of the acute abdomen is highlighted in this article, since it enables both the diagnosis and treatment of broad ligament hernias.
